# Ectodermal dysplasia: a narrative review of the clinical and biological aspects relevant to oral health

**DOI:** 10.3389/fped.2025.1523313

**Published:** 2025-02-27

**Authors:** Ana Carolina Morandini, Oluwatomisin Adeogun, Megan Black, Emily Holman, Kaitlyn Collins, Wesley James, Laura Lally, Ashley Fordyce, Rachel Dobbs, Eve McDaniel, Hannah Putnam, Michael Milano

**Affiliations:** ^1^ Department of Oral Biology & Diagnostic Sciences, Dental College of Georgia at Augusta University, Augusta, GA, United States; ^2^ DMD Program, Dental College of Georgia at Augusta University, Augusta, GA, United States; ^3^ Department of Pediatric Dentistry, Dental College of Georgia at Augusta University, Augusta, GA, United States

**Keywords:** ectodermal dysplasia, tooth development, tooth abnormalities, tooth agenesis, tooth eruption

## Abstract

Ectodermal dysplasias (ED) are disorders that affect ectodermal-derived tissues during embryonic development. These disorders occur when the ectoderm, the outermost layer of embryonic tissue does not develop normally. Patients present abnormalities of two or more ectoderm-derived structures and the clinical presentation can vary greatly depending on the type a patient has. The authors compiled and provided their perspective on articles describing the classification, molecular signaling pathways, systemic and dental implications, genetic diagnosis and dental treatment considerations for patients with ED. Emphasis was placed on the main signaling pathways affecting tooth development and the relevant signs that ED patients can present including dental anomalies. Sources included original or review articles written in English that had an ED focus from PubMed and also information available in National Foundation of Ectodermal Dysplasias website. A broad and flexible narrative review is provided regarding ED which represents a diverse array of systemic symptoms that are often present with dental-related issues. The genetic diagnosis of this condition has evolved significantly during the last decade but is still an adjunct to clinical presentation. The treatment of ED involves a multidisciplinary team encompassing primary care physicians, pediatricians, nutritionists, speech therapists, dental professionals, and geneticists. Evidence from the last decade has significantly expanded our understanding of the classification and molecular signaling pathways involved in the etiology of ED. The dental professional is a critical, essential part of the team of healthcare professionals and often the first step involved in providing personalized and humanistic care and better quality of life to the patients affected by this condition.

## Introduction

Ectodermal dysplasias (ED) are disorders that affect human tissues derived from the ectodermal layer (outermost layer) of embryonic tissue during development. These disorders occur when the ectoderm does not develop normally. Typically, patients are considered for diagnosis with ectodermal dysplasias when two or more ectodermal-derived structures are affected. The skin, hair, teeth, nails, and sweat glands are the most frequently affected tissues typically seen in a patient with ectodermal dysplasia (1). In addition to these tissues, phenotypical features of ectodermal dysplasias can manifest on other ectodermal tissues such as the mammary glands (2), Central Nervous System (CNS), inner and external ear, and ophthalmic (cornea, conjunctiva, lacrimal apparatus) (3).

The abnormal development of ectodermal tissues is typically caused by gene mutations. There are numerous types of ED, and the cause of many of the individual types can be traced back to the mutation of specific genes. (e.g.,EDA1, EDAR, EDARADD, and WNT10A) (4–6). The most common type of mutation causing ED are those that affect the cell signaling process. Because ED encapsulates a very broad array of disorders, the mode of inheritance of ectodermal dysplasia varies. For example, the most commonly occurring type of ectodermal dysplasia is hypohidrotic ectodermal dysplasia (HED), which is a condition transmitted as an X-linked recessive disorder (1).

This narrative review brings a comprehensive summary of the scientific evidence encompassing biological aspects and clinical characteristics of ED. It is the goal of this review to offer a flexible, broad exploration of these conditions highlighting key aspects of molecular signaling pathways that are relevant for the ED-related signs and dental anomalies which are relevant to the dental professional. Sources included PubMed and National Foundation of Ectodermal Dysplasias (NFED) website. The search strategy included articles published in PubMed using keywords “Ectodermal Dysplasia AND symptoms”; “Tooth development AND Ectodermal Dysplasia”; “Molecular signaling AND Tooth Development”; “Ectodermal Dysplasia AND Diagnosis”; “Ectodermal Dysplasia AND Genetics”; “Dentist AND Multidisciplinary Treatment.”

## Classification of the types of ED

There are over 100–200 types of ED resulting in a wide distribution of subgrouping variation and classification systems. The first classification system was created in 1970 by Freire-Maia and is based on a phenotypic classification (7). The phenotypic classification is based on four main structures of the ectoderm that are affected: hair, teeth, nails, and sweat glands. The affected ectodermal structures were used to divide disorders into group A and group B. Group A represents a defect of two or more classical structures. Group B is a defect in one classical structure and at least one of the other ectodermally-derived structures. For example, hypohidrotic ED is classified in Subgroup 1-2-3-4, indicating all four classic ectodermal structures are affected (8). The Freire-Maia research group reported 154 different types of ED in 1994, which was updated in 2001 to include a total of 192 types of ED (7).

Since 1970, there have been major advancements in genomic research and studies on ED. Genome and exome analysis allows diagnosis that is more accurate by considering the genes and signaling pathways involved. In 2017, at the 8th International Conference on Ectodermal dysplasia, a new classification system was implemented (9). With this new system, ED can be more accurately diagnosed when considering affected genes and molecular signaling pathways of genetic inheritance patterns. In the new classification system, ED-related variants of genes are grouped together based on the molecular pathways affected. These genes are involved in numerous molecular pathways linked together in the development of ectodermal transcription factor pathways such as; EDA/NF-*κ*B, Wnt/*β*-catenin or the p63 transcription factor pathways as reviewed elsewhere (9). The enhanced genetic knowledge allowed for the exclusion and reclassification of some disorders previously considered ED. Additionally, it allowed for the consolidation of EDs previously considered to be different types. Despite the advancements in genome and exome research, the knowledge of causative genes for the over 200 types of ectodermal dysplasias remains limited (10).

A more recent study utilized large multicenter databases of electronic health records, such as Oracle Real World Data and established estimated prevalence rates for several of the more common ED syndromes, reporting a total of 49 recognized EDs with molecularly confirmed etiology (11).

Due to the many forms of ED the symptoms are widespread, and disease presentation can vary greatly depending on the patient-type; however common oral symptoms include: cleft lip or palate, thinner than normal enamel, decreased saliva, contributing to increased incidence of caries. The most typical dental abnormalities include hypodontia (absence of 1–6 teeth), microdontia, anodontia (complete absence), abnormal tooth shape, malocclusion and delayed tooth eruption. In ED, there is a characteristic pattern of agenesis that is usually different from the overall population (12). In addition to the oral presentation, patients with ED also tend to have sparse (hypotrichosis), light colored hair or alopecia, abnormal nail thickness, reduction in the number of sweat glands resulting in hypohidrosis (diminished sweating) (13), and eye dryness (14). There is also a classical appearance of periorbital hyperpigmentation in patients with ED along with depressed nasal bridge, malar hypoplasia, and absent or sparse eyebrows and eyelashes (15). There are numerous types of ED with oral signs and symptoms (16). This includes Hypohidrotic ED also known as Christ-Siemens Touraine Syndrome (17). This form of ED is diagnosed through a triad of hypohidrosis or anhidrosis, hypotrichosis and hypodontia. Due to delayed dentition and missing teeth there tends to be hypoplastic alveolar ridges and midface hypoplasia resulting in underdevelopment of the maxilla, reduced volume in the upper jaw, cheekbones and eye socket causing protruding eye appearance. It is also common to see peg shaped or conical teeth and hypodontia or absence of teeth (18).

Ankyloblepharon ED Cleft lip/palate (AEC), also known as Hay wells syndrome, also manifests in the oral cavity. Patients with AEC typically present with cleft lip or palate occurring in most cases and Ankyloblepharon filiforme adnatum (fusion of the eyelids) which can be present near the canthus or corners of the eye or down the midline of the eye (18). Focal Dermal hypoplasia, or Goltz Gorlin syndrome, is characterized by a variety of craniofacial abnormalities including cleft lip, enamel hypoplasia and hypodontia (18) and other enamel defects including microdontia, gemination, fusion, mulberry-like molars, and alveolar notching (19). The most common soft tissue defect is appearance of papillomas in the oral cavity affecting the gingiva, tongue, palate, buccal mucosa and/or pharynx (20). Other ED syndromes worth mentioning include: Incontinentia Pigmenti (IP) and Ectodermal Dysplasia and Immunodeficiency 1: (EDAID1), Odonto-onycho-dermal Dysplasia; (OODD),Schopf-Shulz-Passarge Syndrome, Acro-Dermato-Ungual-Lacrimal Tooth Syndrome(ADULT syndrome), Rapp-Hodgin Syndrome, Limb-Mammary Syndrome(LMS), arthrogryposis and ED, and Dermo-odontodysplasia.

## Molecular signaling pathways involved in tooth development

During 6th week *in utero*, ectoderm derived from the 1st pharyngeal arch forms an epithelium layer which thickens and proliferates forming the dental lamina. Neural crest cells beneath the epithelium surround a core of mesodermal cells. Ectomesenchyme forms from the folding of neural folds. Teeth develop from the complex interactions between oral epithelium and underlying mesenchyme tissue forming the morphology of a developing tooth germ into three stages: a bud, cap, bell stage. Theses epithelial-mesenchymal interactions are a series of programmed, sequential, and reciprocal networks of cell signaling pathways transmitting communications between cells for odontogenesis. Defects in any of these signaling pathways results in arrested tooth and skeletal development (21, 22).

During the initial bud stage of dental development, the dental lamina, develops into tooth buds which protrude into the mesenchyme layer forming the bud shaped enamel organ (which will give rise to the enamel -amelogenesis). During the cap stage, the epithelial bud continues to proliferate. The mesenchymal cells form the dental papilla (future dentin and pulp), the ectomesenchymal cells (derived from the neural crest) condense around the enamel organ and further divide and grow around the enamel organ forming dental follicle (future cementum, periodontal ligament, and adjacent bone). The cap stage consists of outer enamel epithelium (OEE) surrounding the enamel organ, an inner enamel epithelium (IEE) lining the concavity of the enamel organ, and stratum intermedium(SI) adjacent to the IEE, and the remining cell filling the enamel organ are the stellate reticulum (SR). Where the OEE and the IEE join forms the cervical loop forming the future cervix of the tooth. During the cap stage, a localized area of dense cell proliferation near the center of the enamel organ forms the enamel knot. The next stage of the tooth development results in continued growth of the tooth germ which differentiates from the cap to the bell stage. At the junction between the IEE and dental papilla, morpho differentiation forms the future shape of the dental crown. The IEE cells elongate and differentiate into the ameloblasts forming enamel, and the dental papilla differentiate into odontoblasts. Tooth development in both humans and mice is regulated by several signaling centers involving multiple transcription factors and signaling pathways which are reviewed elsewhere (23).

During normal tooth development, although the transition from bud to cap stage is crucial, potential anomalies associated with tooth shape or number at each tooth developmental stage can occur and are outlined in Table 1. The Ectodysplasin (Eda) pathway includes the EDA gene, receptor (EDAR) and adaptor protein (EDARADD) which influences the size of the primary enamel knot and interacts with the Wnt (secreted glycoproteins which help regulate cell determination, migration, polarity, neural patterning, and organogenesis during embryonic development, including tooth morphogenesis of dental epithelium and mesenchyme with odontoblast and ameloblast differentiation) (24) and Fgf pathways. Among the Bone morphogenetic protein (Bmp) family, Bmp4 is also important for promoting the expression of ectodin, which is a Wnt and Bmp antagonist that restricts the expression of the gene Cdkn1a. The gene Cdkn1a is responsible for expressing the cell cycle inhibitor, p21. The protein p21 is necessary for blocking the growth of the enamel knot and the apoptosis of the transient signaling center. Its expression is repressed while the enamel knot is still forming (25). The Wnt, Shh, Bmp, and Fgf pathways are involved in the further growth and shaping of the epithelial tissue into cusps during tooth development (26). The gene Bmp4 stimulates bone formation and tooth development by inducing osteoblastic commitment and differentiation of stem cells which in turn induces transcription factors, Msx 1 and 2 (27).

**Table 1:** Anomalies associated with tooth developmental stages.

Stage	Anomaly
Dental lamina/initiation	Anodontia
Dental lamina/initiation	Hypodontia
Cap	Microdontia
Bell	Abnormal Tooth Shape

During the process of tooth initiation, animal model studies have shown several different signaling molecules (26) are expressed to cause invagination of the dental epithelium, and they are summarized in Table 2. These signals can be epithelial-derived or mesenchymal-derived. The different signaling pathways that are present throughout tooth development, like the Fgf family, Shh, Wnt/B-catenin pathway, and Bmps, have their first appearance during the tooth initiation stage. These pathways play a significant role in the invagination of the dental epithelium in this initial stage. Failure of the above-mentioned signaling pathways to function properly can lead to arrested tooth development at this stage. For example, blocking Fgf pathway during initiation stage disrupts cell migration, resulting in smaller, posterior formed tooth buds (31). The genes Pax9 and Msx1 are also present at this stage and work together to condense mesenchymal tissue (26), and if nonfunctioning, can also lead to the arrest of tooth development (28). Recent discoveries in a murine animal model imply a dynamic expression pattern during tooth development, highlighting the role of Yes associated protein (YAP) and transcriptional coactivator with PDZ-binding motif (TAZ) which control organ development and homeostasis, as reviewed elsewhere (29). The transcription co-activators YAP/TAZ are relevant in cell proliferation, apoptosis, and polarity in the enamel knot region, which affects enamel knot formation, location, and signal release. The dysfunctionality of the enamel knot related to an abnormal YAP/TAZ expression pattern could impair mesenchymal condensing and tooth germ invagination (29).

**Table 2:** Molecular pathways associated with tooth initiation (animal model).

Gene/signaling pathway	Function	Non-functioning gene/signal
Fgf	Regulates size of tooth bud & anterior migration of tooth bud.	Tooth bud forms much smaller and posteriorly.
Shh	Influences the growth and folding of the dental epithelium, guiding the forward movement of the tooth bud.	Growth is arrested, and there is no invagination of dental epithelium.
Wnt/B-Catenin	Epithelial initiator of tooth development and is involved in enamel knot formation.	Development is arrested at Bud stage.
Pax9	Mesenchymal condensation at the Bud stage. Initiates expression of Ffg3 & Ffg10.	Arrested growth
Msx1	Works with Pax-9 to condense mesenchymal tissue at Bud stage. Additionally, Bmp4/Msx-1 pathway suppresses Dkk2 and Sfrp2 (Wnt Antagonists)	Downregulation of Bmp4 and Fgf3 arresting molar development.
Bmp	Works with Fgf to regulate the expression of Pax9 and Msx1.	Not specified but associated with decrease in expression of Msx1.
Activin	Mesenchymal odontogenic signal that is involved in regulating Dkk2 expression	Mandibular molar development halts at the bud stage, while maxillary molars develop normally. It is proposed that differences in activin or Bmp4 expression in maxillary teeth are sufficient to counteract the low levels of Wnt antagonists.
Yap/Taz	May be important for cell proliferation in the dental epithelium and enamel knot formation.	Yap deficiency leads to reduced tooth germ size, while overexpression results in abnormal morphogenesis. Taz may partially compensate for these effects.

References (26–30).

Molecular pathways associated with the subsequent bud to cap transition are depicted in Table 3. During cap and bell stages of tooth development, the proliferation and differentiation of dental stem cells are important for further elongation and invagination of the dental epithelium, and the top five listed genes in Table 4 are involved in this process. Fgf10 is responsible for maintaining the survival of these progenitor cells, and members of the Spry family are responsible for controlling the survival of the cells by negatively regulating Fgf expression (26). The genes Sox2, Pitx2, and Lef1 are also being expressed and are necessary for proper development, differentiation, and proliferation of cells (33). Homeobox genes are also active during the phase of shape-determining pathways (34). In addition to the homeobox genes, the NF-*κ*B pathway is active and affects cusp and enamel formation (35). Lastly, Runx2, which is heavily involved in osteoblast and odontoblast development, is expressed during this stage (36).

**Table 3:** Molecular pathways associated with Bud to Cap transition.

Gene/signaling pathway	Function	Non-functioning gene/signal
Fgf20	A downstream effector of Eda that plays a role in regulating tooth number, size, and shape during morphogenesis.	Effects in tooth formation and morphogenesis.
Eda	Influences the size of the primary enamel knot, regulated by Wnt signaling with Fgf-20 as a downstream effector.	Size of enamel knot is reduced and leads to flattened cusp formation.
Sostdc1 (ectodin)	Its expression is promoted by Bmp4, and it acts as a Wnt and Bmp antagonist. Negative feedback with Bmp4 restricting the expression of Cdkn1.	Knockout results in overexpression of Cdkn1a, along with increased Wnt signaling, leading to the development of supernumerary teeth.
Cdkn1a (p21)	The cell cycle inhibitor p21 restricts the growth and size of the enamel knot and triggers apoptosis in the transient signaling center.	Overexpression of Cdkn1a leads to an enlarged enamel knot and defects in cusp formation.
Wnt, Shh, Bmps, Fgf	They work together to promote the expansion and elongation of adjacent epithelial tissue, leading to cusp formation	Severe defects in tooth number and shape.
Bmp2/4	Primary enamel knot cells express Bmp4 to induce apoptosis in the transient signaling center. Bmp4 also stimulates the expression of ectodin. Furthermore, the BMP4/MSX-1 pathway inhibits the Wnt antagonists Dkk2 and Sfrp2.	Deficiency results in the overexpression of p21 (26). If the Bmp4/Msx-1 pathway is impaired, it fails to suppress the Wnt pathway.
Msx2	Also expressed by primary enamel knot cells to inhibit their growth. It is a homeobox-containing transcription factor.	Effects in the apoptosis of the transient signaling center.
Osr	Antagonizes Msx1 and is crucial for the patterning of molar teeth.	Mice develop supernumerary teeth lingual to their predecessor.

References (23, 26, 28, 32).

**Table 4:** Molecular pathways associated with Cap and bell.

Gene/signaling pathway	Function	Non-functioning gene/signal
Fgf10	Plays a role in the survival of dental epithelial progenitors.	Results in a smaller epithelial layer and loss of ameloblast progenitors.
Spry (Sprouty)	An epithelial negative regulator of FGF signaling.	Loss of this regulator leads to Fgf overexpression and the development of ectopic molars
Sox2	Expressed by dental stem cells, this factor is essential for cell proliferation, differentiation, and development. It inhibits the expression of Pitx2, which in turn restricts the differentiation of dental stem cells.	Loss of this leads to abnormal development of molars and incisors.
Lef-1	Cell proliferation during incisor development.	Overexpression leads to the creation of a new stem cell compartment, while a deficiency results in halted tooth development.
Pitx2	In dental epithelial progenitor cells, the activation of Pitx2, Sox2, and Lef-1 expression is stimulated. Conversely, this expression is suppressed by Sox-2.	Disruptions in the maintenance and proliferation of dental stem cells highlight the need for further research. Such interruptions could have significant implications for tooth regeneration.
Homeobox Genes (Barx1/Dlx1/2 & Msx-1/Msx-2/Alx-3)	Barx1/Dlx1/2 (Molar) and Msx-1/Msx-2/Alx-3 (Incisor) are mesenchyme-derived signals that are important for shape determination and expression is induced in Fgf8 and Bmp4.	Failure of respective teeth to form.
Runx2	These signals may be essential for the differentiation of odontoblasts and osteoblasts, and they are crucial for the transition from the cap stage to the bell stage in tooth development.	Arrests development at the early cap stage.
NF-*κ*B	This pathway is downstream of the EDA/EDAR/EDARADD pathway. NFkB influences enamel and cusp formation in teeth.	Defects in the mineralization of enamel and number of cusps.

References (26, 33–36).

The periodontal ligament and the tooth root develop during the eruption phase, outlined in Table 5. If there is any malfunction during eruption phase, the eruption of the tooth may be delayed or may not occur at all. Through the negative regulation of Shh-Gli1 expression, BMP-Smad4 signaling plays a crucial role in inhibiting Sox2, a crucial factor that suppresses the formation of tooth roots. Moreover, PTHrP (parathyroid hormone-related peptide) aids in the differentiation of cells such as fibroblasts, osteoblasts, and cementoblasts (26). Lastly, Runx2 plays a critical role in this stage because it affects the OPG/RANK/RANKL system, which in turn affects the development of bone apical region of the tooth (33).

**Table 5:** Molecular pathways associated with tooth eruption.

Gene/signaling pathway	Function	Non-functioning gene/signal
Bmp-Smad4	Inhibits Sox2 expression by suppressing the Shh-Gli1 pathway.	HERS is impacted, leading to increased cell proliferation and a lack of cell differentiation. This results in an increased proportion of crown to root.
Parathyroid hormone related peptide	Influences the differentiation of cementoblasts, osteoblasts, and PDL fibroblasts.	Premature differentiation of cementoblasts leads to a defective periodontal ligament and failure of the tooth to erupt.
Sox2	Expression is lost during crown eruption	Root formation will not occur if Sox2 expression is maintained
Shh-Gli1	A regulator of Sox2 expression during crown eruption	Loss of Shh-Gli1 due to Bmp-Smad4 signaling leads to loss of Sox2 expression
Runx2	It acts on the OPG/RANK/RANKL pathway, aiding in the synchronization of tooth development with bone resorption and formation.	Delays the eruption of the tooth.

References (26, 33).

## Molecular signaling pathways in ED-related dental anomalies

In cases of ED, gene mutations often lead to dental anomalies, many of which are linked to key developmental pathways. For example, mutations in genes such as IKBKG (37), NFKBIA (38), and LTBP3 (39) are all involved or interact with the EDA/EDAR/EDARADD/NF-*κ*B pathway, which as mentioned earlier is involved in the formation of the enamel knot and morphogenesis of teeth. Also, TP63 (40), CDH3 (41), KDF1, PORCN (42), KREMEN1 (43), LRP6 (42), and TBX3 (44) are involved with the Wnt/*β*-catenin pathway, which is heavily involved in the initiation of tooth development and morphogenesis. Mutations in TRPS1 can also lead to ectodermal dysplasia with dental anomalies because it interacts with the RUNX2 and OSX pathways involved in the mineralization of teeth. However, a previous study has not been able to replicate differences in tooth number in a mouse model like that observed in the human condition (45). Lastly, TSPEAR mutations have also been found to cause dental anomalies in ED cases, but its function is largely unknown. Based on a study done by Jackson et al. (46), TSPEAR may be involved in the formation of the enamel knot, and when malfunctioning, can result in missing and misshapen teeth.

## Main dental-related signs and systemic manifestations

**Figure 1:**
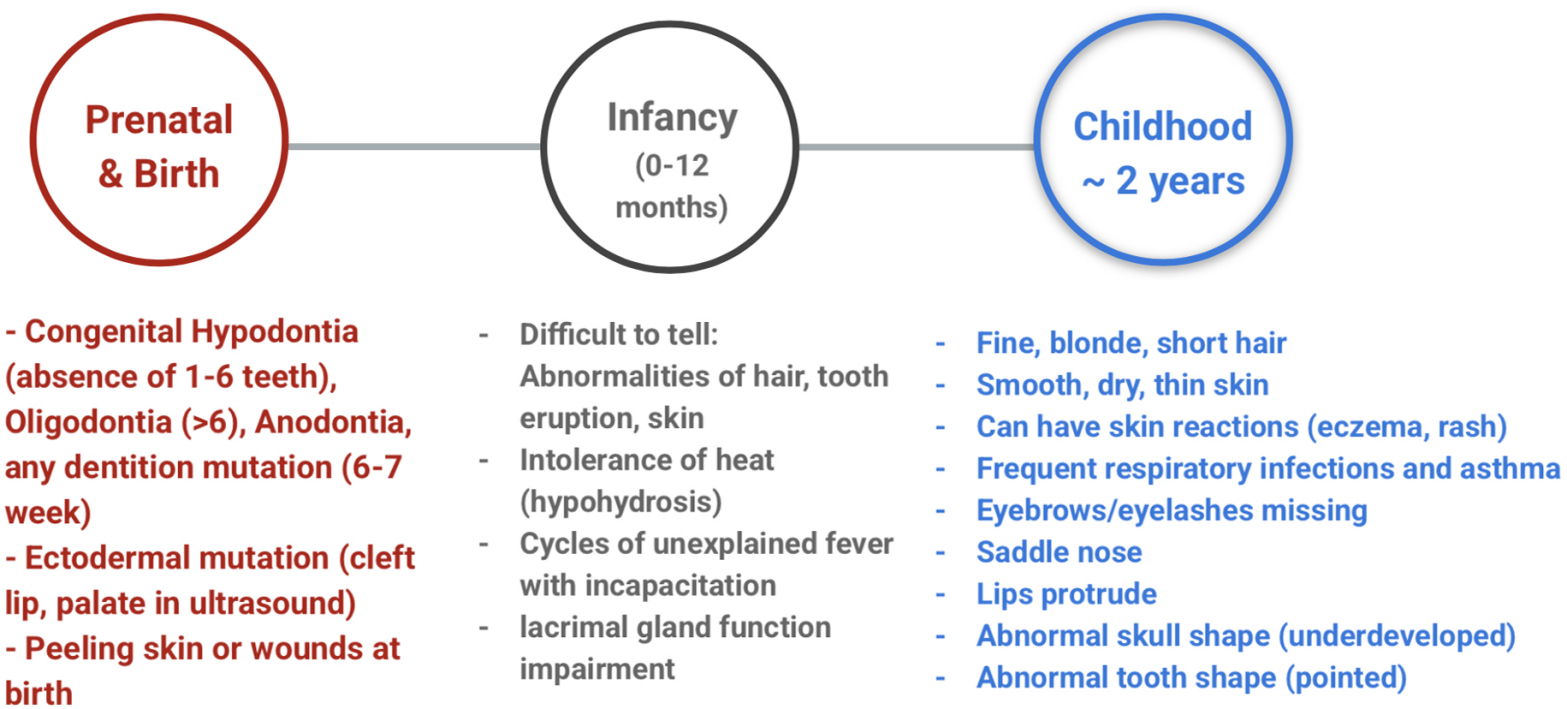
Timeline of perception of main signs and symptoms of ectodermal dysplasia.

The appearance of ED can vary based on the type of ED, the patient's systemic factors, and local factors. Therefore, no one timeline of appearance is congruent to every case of ED. However, there are enough phenotypical appearances between cases to generalize a few prominent signs and symptoms into a timeline featured in Figure 1. Congenital Hypodontia (1–6 teeth), Oligodontia (≥ six teeth), Anodontia, and any dentition mutation happen around the 6th-7th week of embryonic development (47). Ectodermal mutations in non-dental tissues occur around the eighth week of development (8). Certain craniofacial anomalies, such as cleft lip or palate, can be seen in a pre-natal ultrasound. Tooth germ cells can also be seen in an ultrasound to detect if dental mutations affect the number of teeth present (48). At birth, fused or missing limbs or malformation of one or more fingers or toes are seen with certain types of ED such as Ectrodactyly-ectodermal dysplasia, and cleft lip/palate syndrome (EEC) syndrome (49). Specific syndromes such as Rapp-Hodgkin syndrome (50) and ED with skin fragility (51) can present with open head wounds or peeling skin at birth as well.

Within the first year of life, it can be difficult to tell if an infant manifest any of the main signs and symptoms of ED. Many infants with ED have sparse hair, no teeth erupted, or will not show skin abnormalities (8). However, the infants will have cycles of unexplained fever that lead to incapacitation or fatality. This phenomenon happens because the infant displays traits of hypohidrosis, leading to an inability to regulate body temperature (hyperpyrexia) with exertion, producing a fever (52). In addition, the infant can have very few tears when he/she cries because of lacrimal gland function impairment (8). In early childhood, around two years old, many of the characteristic traits of ED can be visible. Children will start manifesting abnormally sparse hair (hypothrichosis), dry/thin skin that can have reactions (eczema, rash), missing/thin eyebrows or eyelashes. The face can look smaller because of the frontal bossing and depression of the nasal bridge (8). The children can also have frequent respiratory infections and asthma. Tooth eruption can be delayed, and the erupted teeth may have an abnormal shape (conical or peg-shaped) and/or an abnormal number (8).

The systemic signs and symptoms expected from patients with ED depends largely on the type of ED of which they are diagnosed. Hypohidrotic is the most common type of ectodermal dysplasia. One would expect to see inability to sweat, sparse hair and common respiratory infections (8). The EDA gene is critical for the interaction between the ectoderm and the mesoderm, which is why skin, sweat glands and nails are disrupted. This can be compared to the IKBKG gene associated with incontinentia pigmenti. IKBKG activates NF-*κ*B, and its highest level of expression is in the CNS, which suggests why seizures are common in patients with incontinentia pigmenti (53, 54).

It is very important to recognize the systemic symptoms as ED can pose a mortality risk, which is highest in the first year of life and remains throughout a patient's childhood. A previous study compared six boys between the ages of 7 and 12 years old with X-linked hypohidrotic ectodermal dysplasia (XLHED) and six boys between the ages of 14 and 18 years old with XLHED during bicycle ergometry and compared with same number of age-matched healthy boys (six control children and six adolescents) (55). The study found XLHED children showed a statistically significantly greater rise of body temperature during workouts and remained elevated longer when compared to the healthy subjects (55). This inability to cool streams the patient's lack of sweat glands and is the reason recognition of systemic symptoms is crucial. The greatest complication from their inability to sweat is the risk of severe hyperthermia. Mortality of HED is highest during the first year of life (56). If heat exhaustion is left unaddressed and the body's temperature continues to rise, it can progress to heatstroke (55). For those with ED, heatstroke may develop rapidly and with fewer warning signs because of the lack of sweating.

## Diagnosis and genetics

The diagnosis of ED is based on clinical signs and symptoms, microscopic examination of prepared biopsy samples, and genetic testing (Table 6). The most common clinical manifestations of the disease are Hypotrichosis, Hypohidrosis, and Hypodontia. While these are the most common findings, ED should be considered where there is unusual differentiation of any tissues or structures that are derived from the ectoderm such as the hair, nails, skin, cornea, or teeth. The oral manifestations are of particular emphasis in mild cases due to other manifestations, such as sparse hair or brittle nails, going unnoticed or misdiagnosed. These cases typically have dysmorphic tooth development of the permanent dentition around age 6. This can cause parents to seek professional care, which will aid in diagnosis. More severe cases are diagnosed at younger ages due to obvious signs and symptoms like hyperpyrexia, cleft lip or palate, or frequent skin peeling or wounds at birth (8).

**Table 6:** Procedures used to establish the diagnosis of ectodermal dysplasia.

Procedure	Source	Comments	Definitive diagnosis
Clinical findings	History Inspection	Clinical findings can be very suggestive of ED and is currently sufficient to yield final diagnosis	Yes, with significant structures and tissues affected, other differential diagnoses can be ruled out
Microscopic	Tissue biopsy Starch-Iodide test	The presence or absence of sweat glands in skin aids in diagnosis	No
Genetic Testing Single gene Targeted panel Exome sequencing Genome sequencing	Blood Saliva Buccal swab gDNA	Positive genetic testing can aid in diagnosis. Negative results do not rule out the disorder due to low sensitivity of tests. Variants of uncertain significance may pose challenges to the application of this procedure	No Current testing is not reliable enough to always confirm diagnosis

The diagnosis of ED in patients with manifestations suggesting the hypohidrotic form of the disease can be supported microscopically from skin biopsy and the identification of dysmorphic or absent eccrine structures (57). This procedure is limited to the forms of ED that are severe enough to result in complete absence of eccrine ducts. There are many forms of the disease that are considered hidrotic where the sweat glands will appear relatively normal. In these cases, other noninvasive clinical tests can assist in determining the ability to produce sweat such as the starch-iodide paper test. A test that shows minimal to no sweat production should support the diagnosis of HED (57).

Genetic testing can be interpreted as being the best way to confirm a positive diagnosis. This is currently debatable. Variants of uncertain significance, disagreement between clinician and laboratory, low sensitivity, and misinterpretation of results all pose significant issues (58–61). Furthermore, the accuracy of genetic testing can differ between types of ED, genes involved, and severity levels (62). It is important to note that, at the time of this study, genetic testing should be considered an adjunct to clinical signs and symptoms when diagnosing ED. There are multiple forms of genetic testing available, such as single gene testing, targeted gene panel, whole exome sequencing, and genome sequencing. The two tests that are most used are targeted gene panels and exome sequencing. Single gene testing is usually too narrow to be diagnostic and genome sequencing is usually too expensive and other tests are sufficiently diagnostic. Most genetic tests involve PCR amplification and are then analyzed using DNA sequencing, microarrays, or gene expression profiling.

ED can manifest in patients through autosomal inheritance, X-linked inheritance, or *de novo* mutations. The pathway of inheritance plays a role in the type of disease and severity. For X-linked forms, there does not appear to be a correlation between the severity of symptoms and the X-chromosome inactivation pattern (63). During pregnancy of suspected carriers, some invasive genetic testing can be performed on the fetus via chorionic villus sampling (64), although the risks and possible complications of this procedure should be clearly explained to the pregnant person and properly taken into consideration relative to the benefits. Following a positive diagnosis of ED, many patients and parents will develop an adverse emotional response to uncertainty about this disease. Genetic counseling can provide many answers and resources such as information on family planning around this condition, research and treatment opportunities. There may be post-natal feeding implications in humans such as breastfeeding complications although the evidence for these comes from murine animal model (65).

Due to the extensive number of variants and clinical findings of ED, the differential diagnosis list will be equally extensive. Disorders and conditions that are not part of the ED family such as meningitis or pachyonychia congenita should also be considered in the diagnostic process (1, 52). Further research is needed to develop a system that incorporates phenotype, genotype, and molecular pathways to diagnose specific conditions more accurately. Topics not discussed in this review include facial recognition of XLHED phenotypes to aid in diagnosis and noninvasive in-utero studies to determine diagnostic effectiveness. These were omitted due to lack of current research.

## Final considerations and available resources

As mentioned earlier, ED manifests with a diverse array of systemic symptoms, outside of dental-related issues. For dentists, it is extremely important to work as a team with other healthcare providers because treatment for ED requires a multidisciplinary approach. The multidisciplinary team involve primary care physicians, pediatricians, nutritionists, speech therapists, dental professionals, and geneticists (66). Age, psychosocial environment, present dentition, oral hygiene, bone volume, jaw growth and development, orthodontics, orthognathic surgery, implants, maintenance, and costs are just a few factors to consider with treatment. A previous study evaluated the effect of complete denture rehabilitation on the jaw growth in individuals with ectodermal dysplasia from an early age to maturity and showed no significant effects in the jaw growth pattern, but improved facial esthetics and masticatory activity (67). In our perspective, a stepwise approach in dental rehabilitation involves careful planning and a staged process to restore oral function, health, and aesthetics in a structured, manageable way. By addressing immediate needs, restoring function, and providing aesthetic enhancements, this approach ensures that the patient's oral health is improved in a logical and effective manner. The success of dental rehabilitation relies on collaboration between the dentist and patient, with ongoing maintenance and follow-up care to ensure long-term oral health.

For children, mainly those with anodontia and oligodontia, early treatment is going to show the greatest outcomes in terms of nutrition, speech development and socialization. In pediatric patients, dentures are typically the standard of care but require continuous monitoring of facial and jaw growth for frequent revisions. For some children that need better retention of the denture, mini-implants can be an option as a provisional treatment until the jaws are completely grown, which is typically around the age of 18–21. It is important to mention that maintaining primary dentition plays a major role in future therapies such as implants that may be indicated later in life. Maintaining the primary dentition can preserve alveolar bone and maintain space for future restorative options, in addition to supporting esthetics. Other options for restoring esthetic and function include composite bonding, veneers and crowns (68). As growth continues, definitive treatment can become an option, which generally includes dental implants due to their success rates (88.5–97.6%) and comfortability for these patients (69). Typically, a pre-implant bone augmentation is standard procedure to add bone volume to areas of tooth agenesis (68) as reviewed elsewhere. The goal of treatment includes providing age-appropriate dentition that optimizes the function of teeth and therefore nutrition, speech, and development. Comprehensive treatment aims to enhance physical, emotional, and psychosocial development for affected individuals.

Access to a range of specialized resources is essential for patients and families affected by ED, in navigating the challenges associated with being diagnosed with this condition. Founded in 1981, the National Foundation for Ectodermal Dysplasias (NFED) is an organization that offers several programs to provide critical support, from medical care and genetic counseling to community networks and educational materials, helping to improve quality of life and foster a sense of understanding and empowerment within the USA. The NFED (70) provides education and one-on-one support to an individual and family who have been diagnosed with ED in learning about the condition, what to expect, and what other NFED resources there are to help. The Family Liaison program comprised of adults with ED and parents of affected children volunteer to share the experiences and offer empathy with personal support and guidance. Most significantly, the NFED hosts Family Conferences, as well as supports regional conferences, where families can meet others affected by ED, share and hear others’ experiences, create a support network, learn from expert doctors and dentists and receive free dental consultation.

According to the NFED website (70), a person with ED can expect a very expensive dental rehabilitation in a lifetime, so the programs intended to alleviate some of the financial burden are of great benefit to individuals and their families. The NFED has a network of Dental Treatment Centers throughout the United States to aid families in finding dental providers who are suited to treat the complexities of ectodermal dysplasia. Recognizing that cost can be a barrier to getting needed treatments and services associated with an ED diagnosis, the Treatment Assistance Program funds eligible individuals for numerous services and items. A previous study (71) demonstrated the significance of the psychological burden of oligodontia, highlighting the importance of a holistic approach by healthcare professionals.

When multiple missing teeth and/or conical teeth are encountered, the dental professional should investigate other signs and symptoms of ED and must not forget that the dentist may be the first step in the diagnosis of this genetic condition.
